# Serum LRG1 as a non-invasive biomarker for identifying complex coronary anatomy in pre-angiography risk stratification

**DOI:** 10.3389/fcvm.2026.1818245

**Published:** 2026-06-04

**Authors:** Zhengkai Yang, Zirui Liu, Ying Xu, Haocheng Wang, Yu Lu, Cao Zou

**Affiliations:** 1 Cardiology Department, Hulunbuir People's Hospital, Hulunbuir City, Inner Mongolia Autonomous Region, China; 2 Cardiology Department, First Affiliated Hospital of Soochow University, Suzhou, Jiangsu Province, China

**Keywords:** biomarker, coronary artery disease, LDL-C, LRG1, SYNTAX score

## Abstract

**Background:**

The SYNTAX Score provides a quantitative evaluation of the complexity of coronary anatomy and informs revascularization strategies; however, its reliance on invasive angiography limits its use in early non-invasive risk assessment. Leucine-rich alpha-2 glycoprotein-1 (LRG1), a novel serum biomarker associated with inflammation and vascular injury, has an unclear relationship with the SYNTAX Score. This study aims to investigate whether LRG1 is an independent risk factor for complex coronary lesions. Furthermore, we explored if a combined model with LDL-C could improve predictive performance.

**Methods:**

Bioinformatics analyses were conducted on GEO datasets (GSE60993, GSE62646) to compare stable coronary artery disease (SCAD) with ST-elevation myocardial infarction (STEMI), as well as healthy individuals with acute myocardial infarction (AMI) patients. Serum levels of LRG1 were quantified using enzyme-linked immunosorbent assay (ELISA). Statistical approaches, including least absolute shrinkage and selection operator (LASSO) regression and Boruta feature selection, were employed to identify independent risk factors, which were subsequently incorporated into a logistic regression model. The diagnostic performance of the model was evaluated using receiver operating characteristic (ROC) curve analysis, calibration tests, and decision curve analysis.

**Results:**

GEO database analysis revealed significant overexpression of LRG1 in patients with AMI. This single-center observational study included 138 patients with confirmed coronary artery disease (CAD). Serum LRG1 levels were significantly elevated in AMI patients compared to non-AMI patients. Furthermore, individuals with SYNTAX Scores ≥ 23 demonstrated higher serum LRG1 concentrations than those with Scores < 23 [277.2 (122.8–393.6) μg/mL vs. 113.4 (65.8–248.9) μg/mL, *P* < 0.001]. Feature selection using the Boruta algorithm and LASSO regression identified LRG1 and low-density lipoprotein cholesterol (LDL-C) as independent predictors of elevated SYNTAX Score ≥ 23. The logistic regression model, depicted as a nomogram, exhibited moderate discriminatory ability (AUC = 0.771, 95% CI: 0.685–0.856), excellent calibration (Hosmer-Lemeshow test, *P* = 0.416), and demonstrated clear clinical net benefit.

**Conclusions:**

This study provides preliminary evidence that serum LRG1, in conjunction with LDL-C, may serve as a non-invasive adjunct for identifying CAD patients with complex coronary anatomy (SYNTAX Score ≥23).

## Introduction

The management of CAD has shifted from a one-size-fits-all approach to precision medicine, emphasizing the anatomical complexity of coronary lesions. Within this context, SYNTAX Score has emerged as the principal quantitative tool for assessing coronary anatomy (1). It is widely regarded as the gold standard for risk stratification and plays a critical role in guiding clinical decisions between percutaneous and surgical revascularization strategies. However, the reliance of the SYNTAX Score on invasive coronary angiography limits its utility for early, non-invasive risk evaluation, highlighting the need for biomarkers capable of predicting complex coronary pathology prior to angiographic assessment.

Unlike traditional imaging techniques, serological biomarkers provide a non-invasive and readily accessible method for patient evaluation. Although numerous inflammatory biomarkers have been associated with CAD severity, most are acute-phase reactants with low specificity for vascular remodeling. Recently, LRG1 has been identified as a promising novel biomarker. LRG1 is unique in that it directly modulates TGF-*β* signaling and promotes pathological angiogenesis—processes central to atherosclerotic plaque progression and instability (2). Its upregulation in inflammatory conditions contributes to vascular endothelial injury and dysfunction (3, 4). For example, Sharon et al. conducted a study involving 2,058 diabetic patients and reported significantly elevated plasma LRG1 levels in those with peripheral vascular disease (5), thereby linking increased LRG1 concentrations to a greater risk of vascular complications. These findings emphasize the clinical relevance of LRG1 in vascular disorders. We therefore hypothesized that LRG1 might reflect not only inflammatory burden but also the anatomical complexity captured by the SYNTAX Score.

Despite these advances, a thorough and systematic investigation into the relationship between circulating LRG1 levels and the SYNTAX Score has yet to be performed. Moreover, the potential of LRG1 as a diagnostic biomarker for identifying patients with high SYNTAX Scores remains unexplored. Addressing this knowledge gap is crucial for the effective translation of biomarker research into clinical practice. The intended clinical application of LRG1 is as a pre-angiography risk stratification tool. Identifying patients with a high likelihood of complex coronary lesions (SYNTAX Score ≥ 23) before invasive angiography could help prioritize catheterization schedules, guide pre-procedural planning, and optimize the use of healthcare resources. This would complement, not replace, the angiographic SYNTAX score.

## Materials and methods

### Study design and participants

This single-center observational study enrolled 180 patients with suspected coronary artery disease (CAD) admitted to the Department of Cardiology at the First Affiliated Hospital of Soochow University from November 2023 to January 2024. After initial screening, 138 patients with CAD confirmed by coronary angiography were included in the analysis. The cohort included 100 males and 38 females, with a mean age of 63.5 ± 11.9 years. Inclusion criteria required participants to be 18 years or older with confirmed CAD. Exclusion criteria included severe cerebrovascular, hepatic, renal, infectious, autoimmune, hematological disorders, malignancies, and other cardiomyopathies. The study was approved by the hospital Ethics Committee (Approval No. [2024] 230). Given the retrospective design and use of de-identified samples, informed consent was waived.

The complexity of CAD was evaluated using the SYNTAX Score (6), a validated angiographic tool. Two experienced interventional cardiologists, blinded to patients' clinical data and outcomes, independently reviewed each patient's coronary angiography images to determine the Score. Based on the SYNTAX Score, patients were divided into two subgroups: (i) low SYNTAX Score (<23) and (ii) high SYNTAX Score (≥23).

### Bioinformatics analysis

For bioinformatics analysis, datasets of patients with stable coronary artery disease (SCAD) and acute myocardial infarction (AMI) were obtained from the Gene Expression Omnibus (GEO) database. Two independent datasets, GSE62646 (7) and GSE60993 (8), were analyzed separately. GSE62646 contains RNA sequencing data from peripheral blood mononuclear cells of 14 SCAD and 28 ST-segment elevation myocardial infarction (STEMI) patients. GSE60993 includes whole peripheral blood RNA sequencing data from 7 healthy controls and 7 STEMI patients. After confirming dataset integrity, differential gene expression analysis was performed.

Principal component analysis (PCA) assessed sample clustering in gene expression data, visualized using the R package quality control, normalization and differential expression analysis were performed with the “limma” package, using thresholds of |log₂ fold change| ≥ 0.5 and adjusted *P*-value < 0.05. Differentially expressed genes (DEGs) were displayed in volcano plots, highlighting LRG1 for its potential role in AMI pathophysiology.

### Serum sample collection and LRG1 detection

Peripheral blood samples from CAD patients were allowed to clot at room temperature, then centrifuged at 1,000 × g for 20 min at 4 °C within two hours of collection. Serum was aliquoted into 2 mL Eppendorf tubes and stored at −30 °C. LRG1 levels were measured using a commercial ELISA kit (JL14662, Shanghai Jianglai Biological) with a detection range of 25–800 μg/mL and a lower limit of 1.0 μg/mL. Blood samples were drawn within 24 h of hospital admission, prior to invasive angiography. Fasting status was not uniformly enforced due to the inclusion of emergency admissions; however, LRG1 has not been reported to be significantly affected by prandial state. The ELISA kit had an intra-assay coefficient of variation (CV) of <8% and an inter-assay CV of <10%, according to the manufacturer's specifications. All samples were measured in duplicate, and the mean value was used for analysis.

### Statistical analysis

In this exploratory study, 28 clinical and laboratory variables were initially considered. Given this large variable pool and a limited number of outcome events (*n* = 25), rigorous feature selection was crucial to avoid overfitting. Therefore, both the Boruta algorithm and LASSO regression were employed to perform data-driven variable reduction. These methods, which apply stringent penalties and permutation-based filtering, successfully reduced the 28 candidate variables to two highly retained features: LRG1 and LDL-C. The final prediction model was then developed using these two predictors. The sample size adequacy for this final model was assessed using the events-per-variable (EPV) criterion. With 25 events for the primary outcome (SYNTAX Score ≥23) and 2 predictors in the model, the EPV was 12.5. This exceeds the commonly recommended minimum of 10, suggesting an acceptable risk of overfitting for the final two-predictor model. To further mitigate over-optimism from the small event count, the final model's discrimination and calibration were internally validated using 1,000 bootstrap resamples. Variables with a missing data rate that exceeded 30% were excluded from the analysis, while for variables with missing data rates below 30%, multiple imputation was applied. Statistical analyses were performed as follows: Normally distributed continuous variables were presented as mean and standard deviation $\left(\bar{x}\pm \;s\right)$ and compared using t-test. Non-normally distributed variables were presented in median and quartile and compared with nonparametric test. Categorical variables were reported as percentage (%) and analyzed using chi-square test.

Feature selection was performed using the least absolute shrinkage and selection operator (LASSO) regression and Boruta analysis. Statistically significant variables (*p* < 0.05) were identified as independent risk factors, and included in the final logistic regression model. Then, a corresponding nomogram was plotted. The model's diagnostic performance was evaluated using receiver operating characteristic (ROC) curves, while calibration and clinical utility were assessed via the Hosmer–Lemeshow test, calibration curve, and decision curve analysis (DCA). To account for potential over-optimism in model performance, internal validation was performed using 1,000 bootstrap resamples. All statistical analyses were performed using R 4.5.2 (https://www.r-project.org). A two-sided *p*-value of <0.05 was considered statistically significant.

## Results

### Bioinformatics analysis

**Figure 1:**
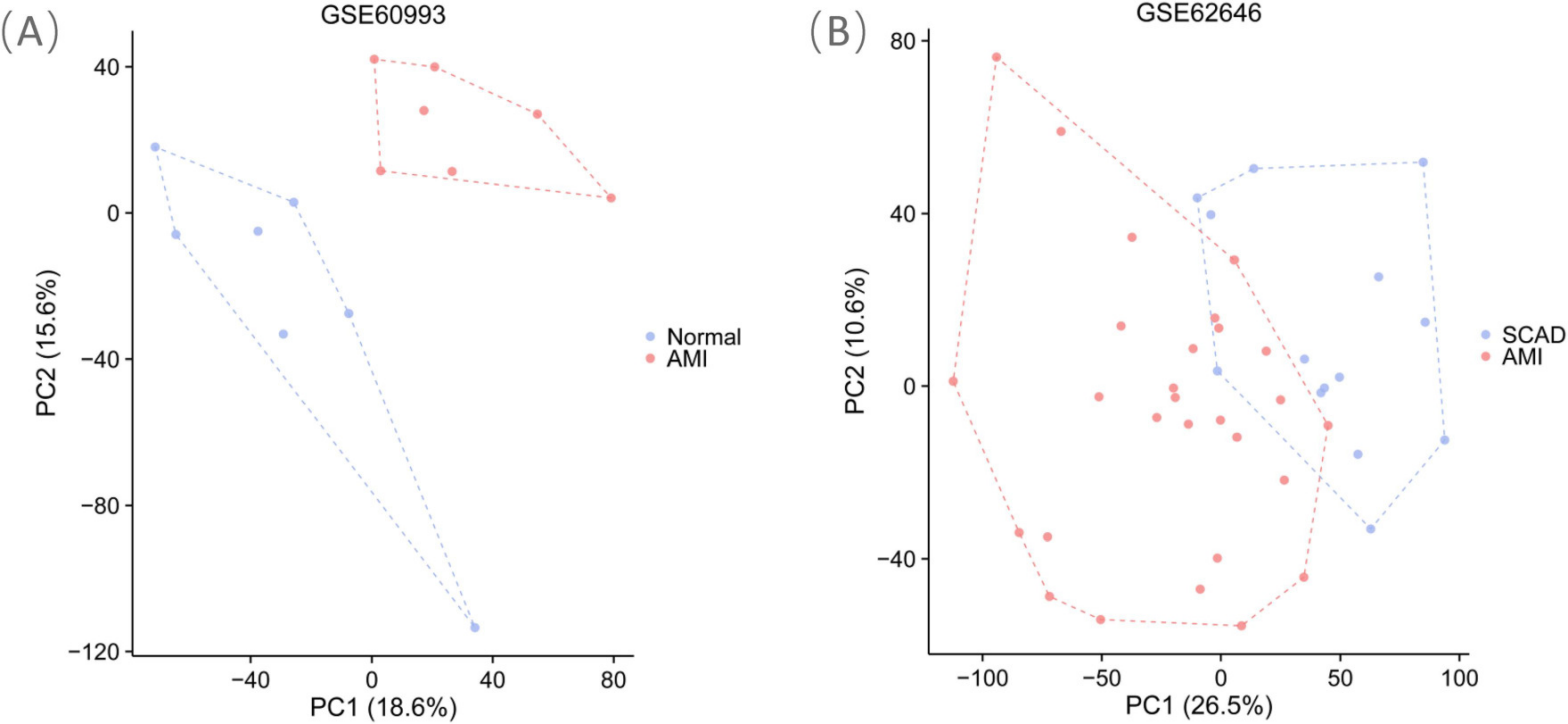
PCA of gene expression profiles in GSE60993. **(A)** and GSE62646 **(B)** datasets.

The data quality was assessed using principal component analysis (PCA). The PCA plot of the GSE60993 dataset clearly separated AMI patients from controls, while the GSE62646 plot showed distinct clusters for AMI and SCAD patients. These results confirm the data's reliability for further analysis (Figure 1).

**Figure 2:**
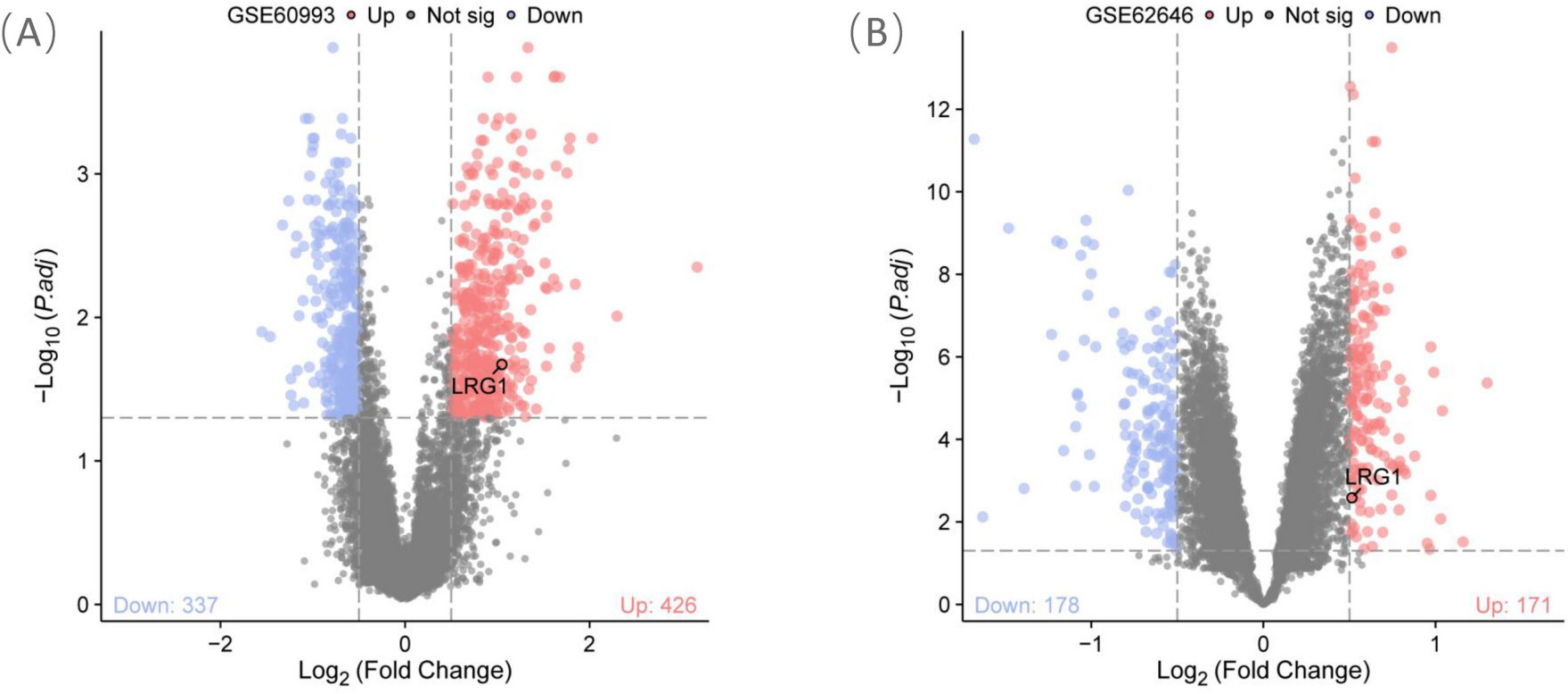
Volcano plots showing differentially expressed genes in AMI vs. control/SCAD. **(A)** Volcano plot of dataset GSE60993 **(B)** Volcano plot of dataset GSE62646.

In GSE60993, 426 genes were upregulated and 337 downregulated in AMI patients compared to controls, including a significant upregulation of LRG1 (log₂FC = 1.05, adj. *P* = 0.02). Similarly, in GSE62646, 171 genes were upregulated and 178 downregulated in AMI patients compared to SCAD, including a significant upregulation of LRG1 (log₂FC = 0.514, adj. *P* = 0.03) (Figure 2). These bioinformatic analyses establish that LRG1 is significantly upregulated during acute myocardial infarction, a condition intimately linked to complex coronary pathology. This serves as the molecular premise for our subsequent clinical investigation, which directly examines the relationship between serum LRG1 levels and angiographically determined coronary lesion complexity.

### Characteristics of the study cohort

**Figure 3:**
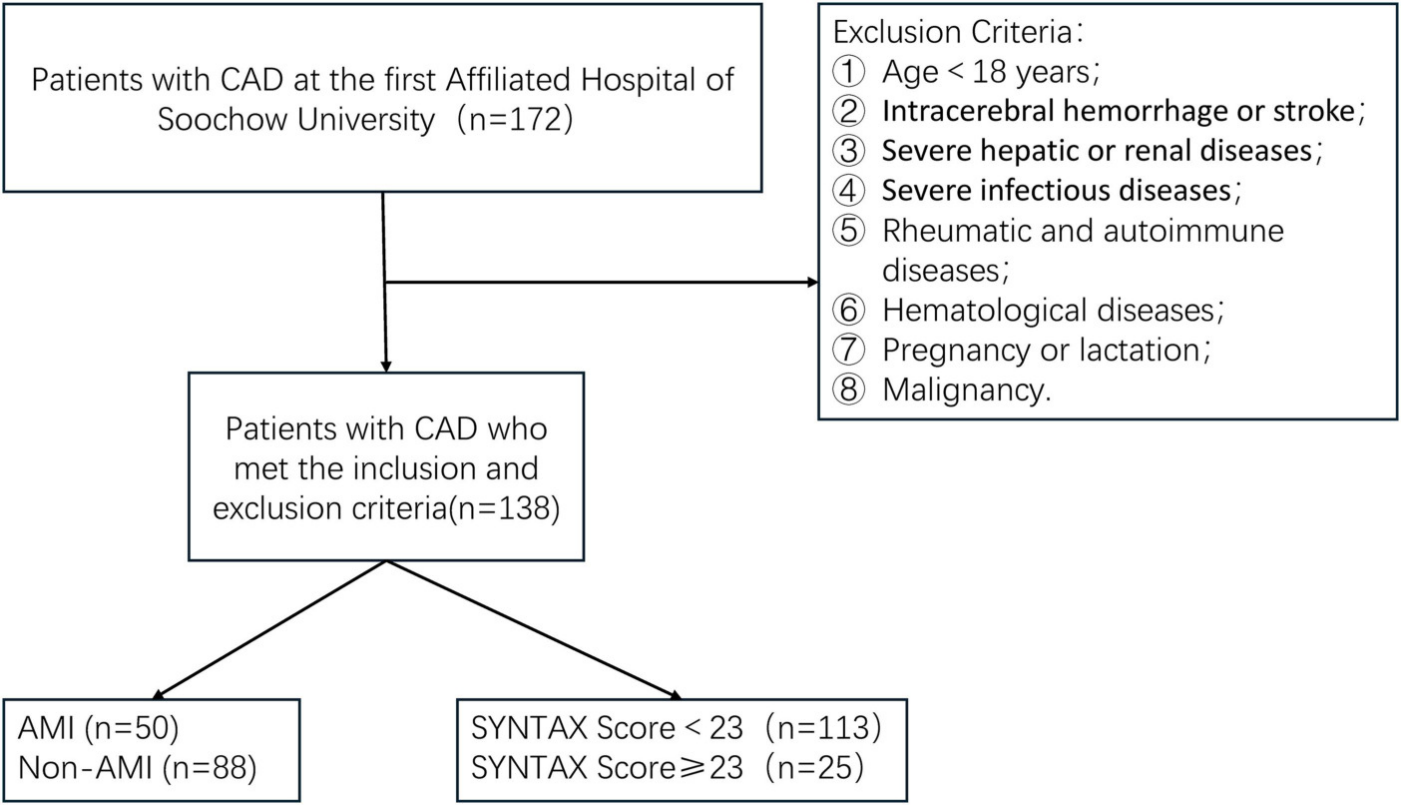
The study flowchart.

Based on inclusion and exclusion criteria, 138 CAD patients from the First Affiliated Hospital of Soochow University were enrolled for analysis (Figure 3). Baseline characteristics did not differ significantly between the non-AMI and AMI groups. However, the AMI group had significantly higher serum LRG1 levels [277.2 (122.8–393.6) μg/mL vs. 113.4 (65.8–248.9) μg/mL, *P* < 0.001] and a greater proportion with SYNTAX Scores ≥ 23 [28.0% vs. 12.5%, *P* < 0.05] (Table 1). Similarly, patients with SYNTAX Scores ≥ 23 had higher serum LRG1 levels than those with Scores < 23 [309.2 (166.0–391.7) μg/mL vs. 122.9 (73.9–278.6) μg/mL, *P* < 0.001] (Table 2).

**Table 1:** Comparison of baseline clinical characteristics between the AMI and non-AMI groups.

Characteristic	NAMI Group (*n* = 88)	AMI Group (*n* = 50)	*P*
Age(years)	63.3 ± 11.25	63.7 ± 13.1	0.861
Gender			
Male [*n*, (%)]	60 (68.2%)	39 (78.0%)	0.218
Female [*n*, (%)]	28 (31.8%)	11 (22.0%)	
BMI(kg m^−2^)	24.8 ± 3.14	25.3 ± 3.17	0.379
Smoking [*n*, (%)]	24 (27.3%)	19 (38.0%)	0.191
HTN [*n*, (%)]	61 (69.3%)	35 (70.0%)	0.933
DM [*n*, (%)]	30 (34.1%)	22 (44.0%)	0.248
HF [*n*, (%)]	18 (20.5%)	36 (72.0%)	<0.001
LAD(mm)	40.7 ± 4.89	40.7 ± 5.42	0.998
LVEF(%)	56.3 ± 14.1	44.8 ± 12.96	<0.001
LVD(mm)	50.5 ± 8.08	52.9 ± 7.47	0.083
LVS(mm)	35.8 ± 10.45	41.2 ± 49.30	0.003
SYNTAX Score≥23 [*n*, (%)]	11 (12.5%)	14 (28.0%)	0.023
Blood indicators			
WBC(*10^9/L)	6.9 ± 1.91	8.7 ± 3.26	<0.001
LYM(*10^9/L)	1.7 ± 0.74	1.3 ± 0.63	0.005
MO(*10^9/L)	0.4 ± 0.13	0.6 ± 0.28	0.001
NE(*10^9/L)	4.6 ± 1.79	6.7 ± 2.94	<0.001
PLT(*10^9/L)	204.3 ± 56.29	221.1 ± 79.54	0.15
HB（g/L）	135.0 ± 15.3	153.7 ± 144.5	0.23
ALT(U/L)	27.1 ± 35.31	37.9 ± 24.07	0.056
TB(umol/L)	17.5 ± 14.34	15.8 ± 7.70	0.447
TG(mmol/L)	1.4 ± 0.85	1.7 ± 1.12	0.101
LDL-C(mmol/L)	2.3 ± 1.09	3.1 ± 2.94	0.018
HDL-C(mmol/L)	1.0 ± 0.26	0.9 ± 0.24	0.054
UA(umol/L)	357.7 ± 101.74	419.0 ± 104.32	0.001
GLU(mmol/L)	5.8 ± 1.55	6.7 ± 2.63	0.006
eGFR(mL/min)	90.3 ± 22.59	72.2 ± 25.60	<0.001
CRP(mg/L)	1.7 (0.7, 3.7)	7.0 (2.2, 31.3)	<0.001
CTnT(pg/mL)	9.6 (6.2, 17.9)	187.1 (68.7, 1,247.3)	<0.001
NT-proBNP(pg/mL)	84.1 (34.0, 367.6)	1,798.5 (404.7, 3,606.5)	<0.001
LRG1(ug/mL)	113.4 (65.8, 248.9)	277.2 (122.8, 393.6)	<0.001

HTN, hypertension; DM, diabetes mellitus; HF, heart failure; LAD, left atrial diameter; LVEF, left ventricular ejection fraction; LVD, left ventricular end-diastolic dimension; LVS, left ventricular end-systolic dimension; WBC, white blood cell; LYM, lymphocyte; MO, monocytes; NE, neutrophil; PLT, Platelet; HB, hemoglobin; ALT, alanine aminotransferase; TB, total bilirubin; TG, triglyceride; LDL-C, low-density lipoprotein cholesterol; HDL-C, high density lipoprotein cholesterol; UA, urinary acid; GLU, glucose; eGFR, estimated glomerular filtration rate; CRP, c-reactive protein; CTnT, cardiac troponin t; NT-proBNP, n-terminal pro-b-type natriuretic peptide; LRG1, leucine-rich *α*-2 glycoprotein-1.

**Table 2:** Comparison of baseline clinical characteristics between the SYNTAX score <23 and ≥23 groups.

Characteristic	SYNTAX Score＜23 Group (*n* = 113)	SYNTAX Score ≥23 Group (*n* = 25)	*P*
Age(years)	63.4 ± 12.01	63.6 ± 11.41	0.935
Gender			
Male [*n*, (%)]	80 (70.8%)	19 (76.0%)	0.601
Female [*n*, (%)]	33 (29.2%)	6 (24.0%)	
BMI(kg m^−2^)	24.7 ± 3.05	26.3 ± 3.34	0.027
Smoking [*n*, (%)]	37 (32.7%)	6 (24.0%)	0.393
Hypertension [*n*, (%)]	79 (69.9%)	17 (68.0%)	0.851
Diabetes Mellitus [*n*, (%)]	38 (33.6%)	14 (56.0%)	0.037
Heart Failure [*n*, (%)]	37 (32.7%)	17 (68.0%)	0.001
LAD(mm)	40.6 ± 4.92	41.2 ± 5.10	0.561
LVEF(%)	53.2 ± 14.73	44.7 ± 14.60	0.01
LVD(mm)	51.6 ± 8.16	51.8 ± 7.56	0.906
LVS(mm)	37.3 ± 10.48	39.8 ± 9.67	0.281
Blood indicators			
WBC(*10^9/L)	7.3 ± 2.56	8.8 ± 2.67	0.01
LYM(*10^9/L)	1.6 ± 0.74	1.5 ± 0.66	0.722
MO(*10^9/L)	0.5 ± 0.17	0.6 ± 0.32	0.025
NE(*10^9/L)	5.1 ± 2.40	6.6 ± 2.52	0.006
PLT(*10^9/L)	204.4 ± 58.39	237.7 ± 89.03	0.022
HB（g/L）	142.4 ± 96.58	138.6 ± 19.93	0.843
ALT(U/L)	27.7 ± 18.48	45.7 ± 63.16	0.011
TB(umol/L)	16.7 ± 13.24	17.6 ± 7.19	0.73
TG(mmol/L)	1.6 ± 1.02	1.4 ± 0.60	0.349
LDL-C(mmol/L)	2.3 ± 1.04	3.8 ± 3.97	<0.001
HDL-C(mmol/L)	1.0 ± 0.26	0.9 ± 0.26	0.509
UA(umol/L)	369.4 ± 99.71	427.5 ± 124.34	0.013
GLU(mmol/L)	5.9 ± 1.92	7.2 ± 2.36	0.004
eGFR(mL/min)	84.8 ± 24.94	79.0 ± 26.30	0.299
CRP(mg/L)	1.7 (0.7, 3.7)	7.0 (2.2, 31.3)	0.036
CTnT(pg/mL)	9.6 (6.2, 17.9)	187.1 (68.7, 1,247.3)	0.039
NT-proBNP(pg/mL)	84.1 (34.0, 367.6)	1,798.5 (404.7, 3,606.5)	0.005
LRG1(ug/mL)	122.9 (73.9, 278.6)	309.2 (166.0, 391.7)	<0.001

HTN, hypertension; DM, diabetes mellitus; HF, heart failure; LAD, left atrial diameter; LVEF, left ventricular ejection fraction; LVD, left ventricular end-diastolic dimension; LVS, left ventricular end-systolic dimension; WBC, white blood cell; LYM, lymphocyte; MO, monocytes; NE, neutrophil; PLT, Platelet; HB, hemoglobin; ALT, alanine aminotransferase; TB, total bilirubin; TG, triglyceride; LDL-C, low-density lipoprotein cholesterol; HDL-C, high density lipoprotein cholesterol; UA, urinary acid; GLU, glucose; eGFR, estimated glomerular filtration rate; CRP, c-reactive protein; CTnT, cardiac troponin t; NT-proBNP, n-terminal pro-b-type natriuretic peptide; LRG1, leucine-rich *α*-2 glycoprotein-1.

### Selection of predictive variables, model construction, and model evaluation

#### Selection of predictive variables and model construction

**Figure 4:**
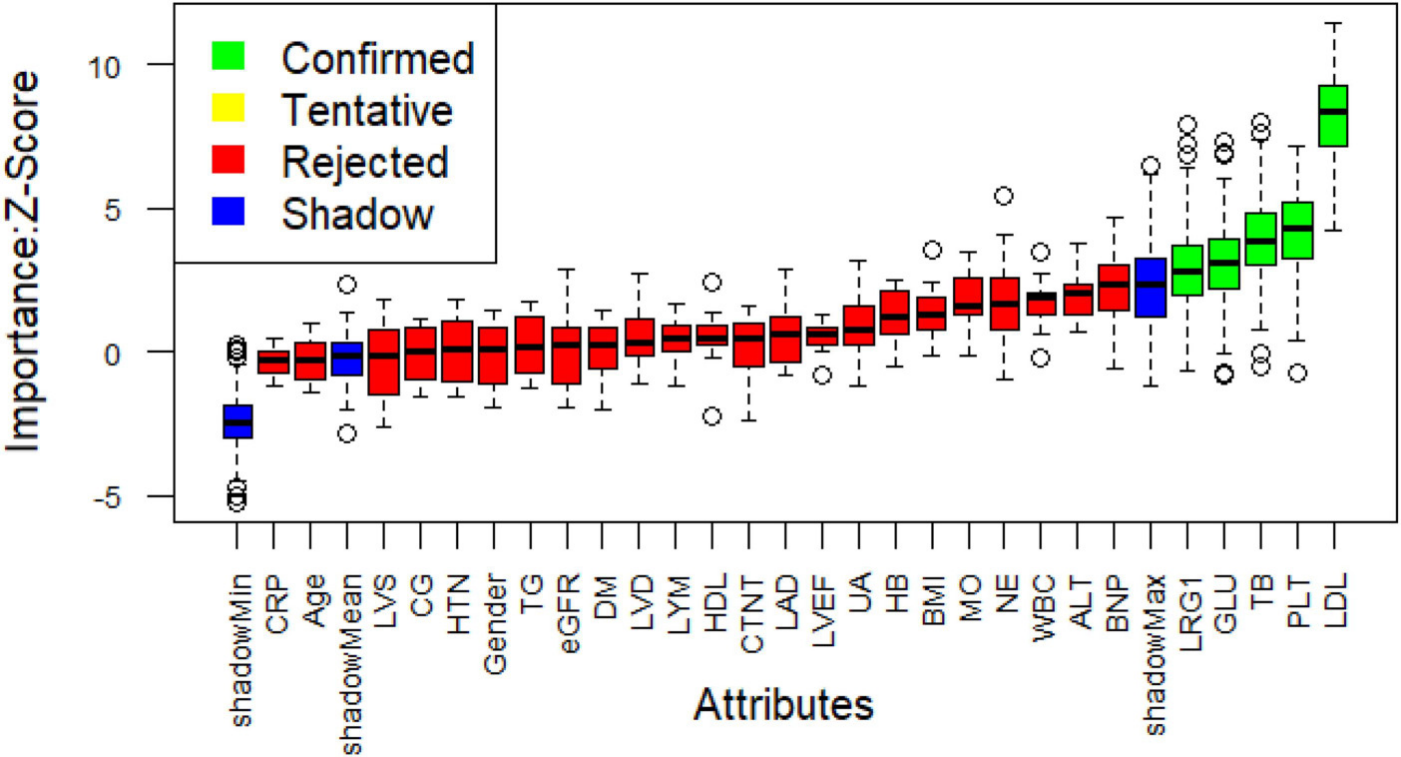
Feature selection using the boruta algorithm categorizes features as follows: green indicates clinically significant features retained to enhance predictive performance; red marks statistically insignificant features excluded from further analysis; yellow denotes borderline features needing further investigation; blue represents shadow features (permuted variables) used for benchmarking but excluded from model training.

**Figure 5:**
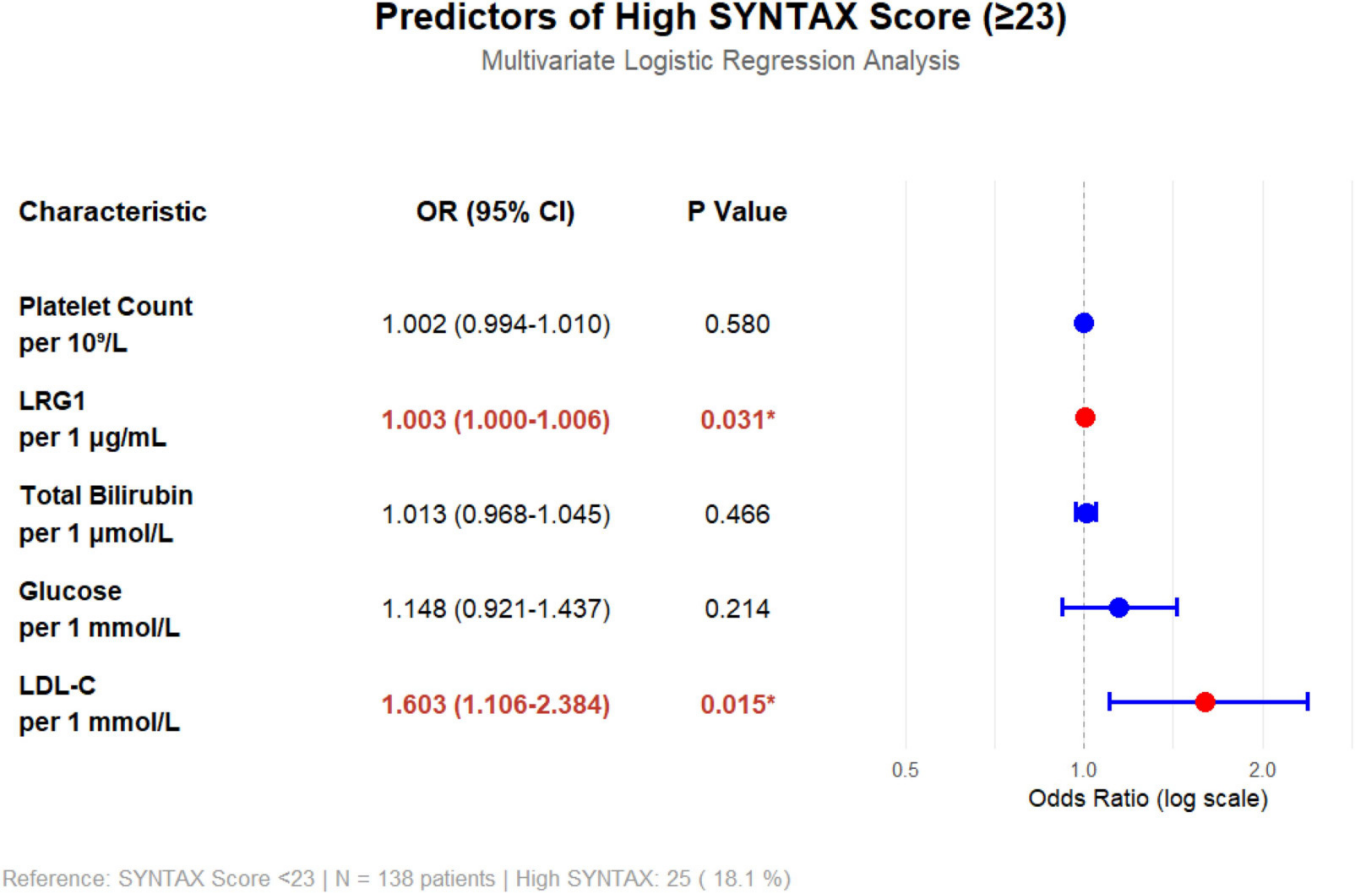
Multivariable logistic regression analysis of SYNTAX score ≥23 in CAD patients.

**Figure 6:**
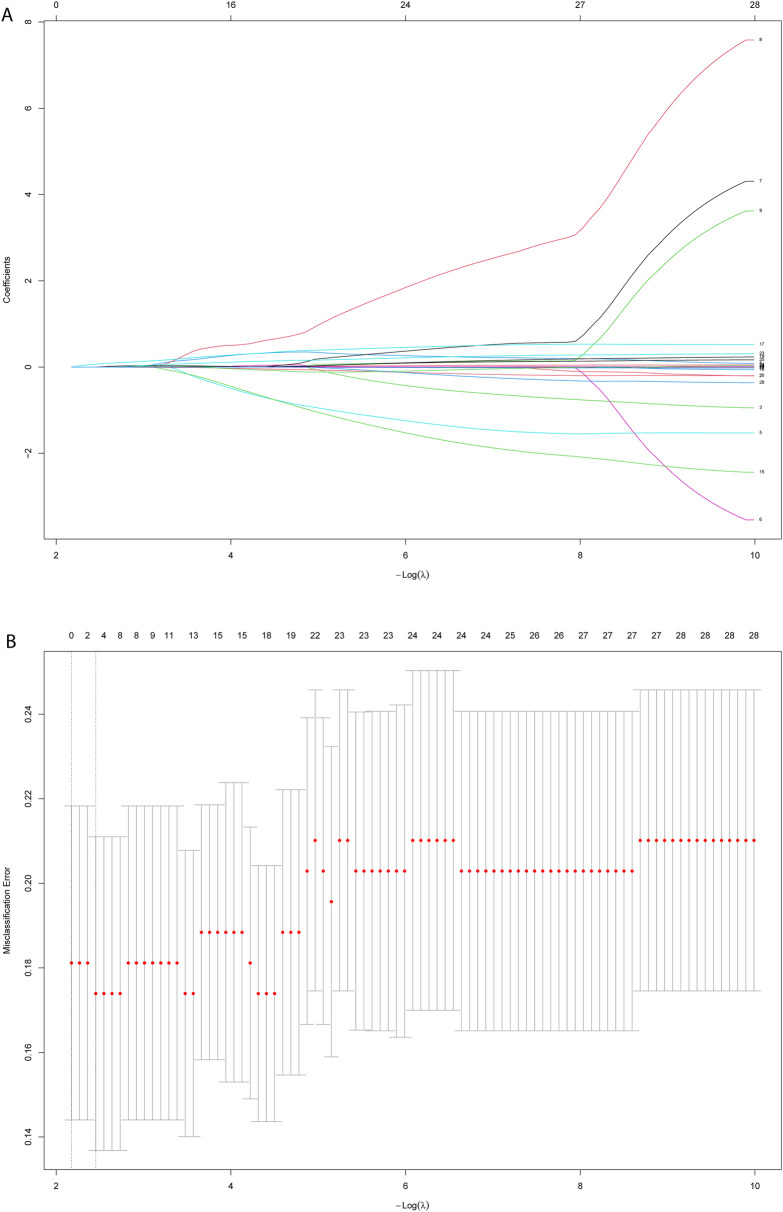
Screening of variables based on lasso regression. **(A)** The variation characteristics of the coefficients of the variables. **(B)** The selection of the optimal value of the parameter *λ* in the Lasso regression model using the cross-validation method.

**Figure 7:**
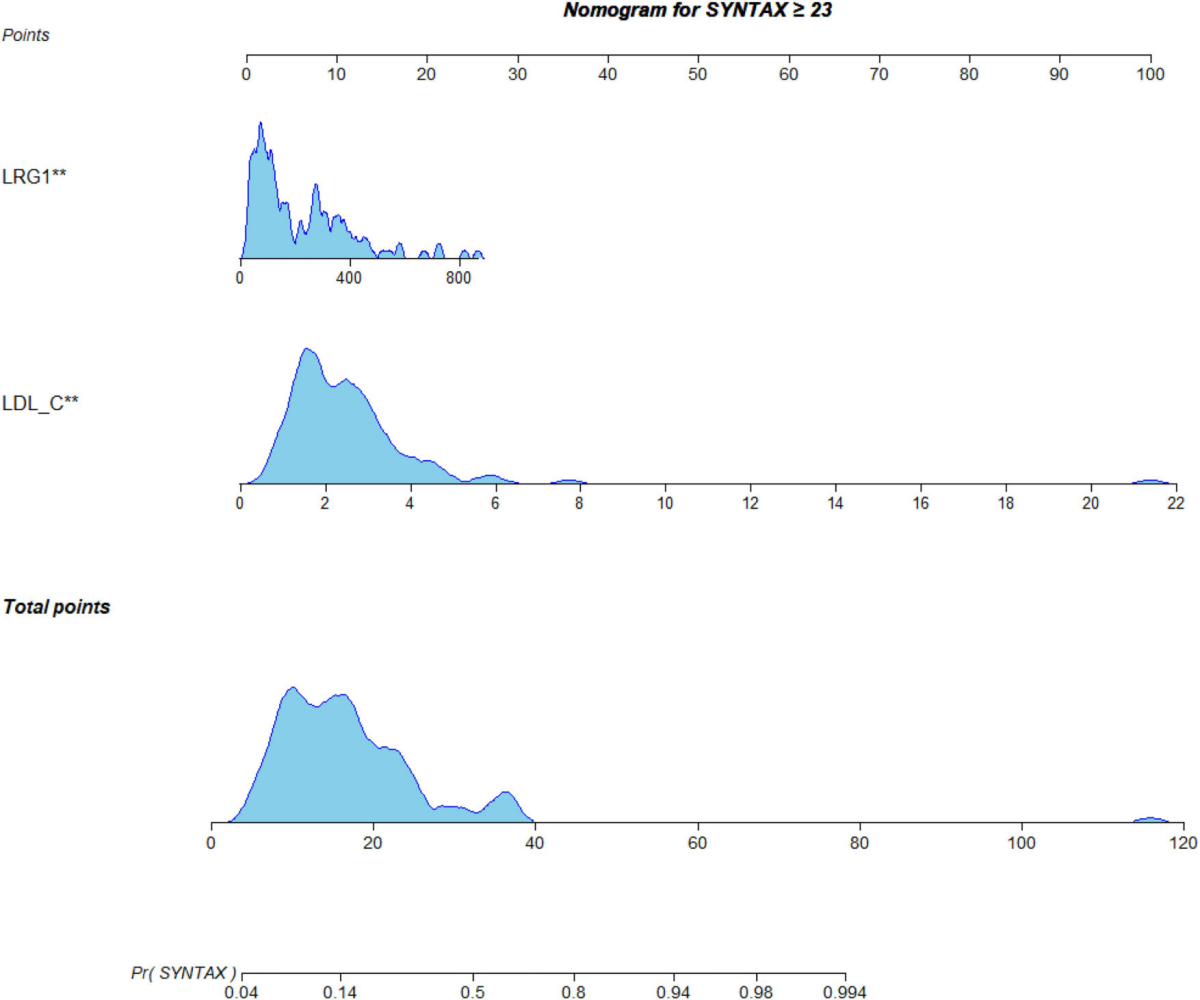
Nomogram for predicting the SYNTAX score ≥23 in CAD patients.

The Boruta algorithm initially identified five significant predictive variables: LRG1, PLT, GLU, TB, and LDL-C (Figure 4). A multivariable logistic regression incorporating these factors (Figure 5) was used to build the final prediction model, visually represented as a nomogram. LASSO regression further refined the selection to two key factors, LRG1 and LDL -C (Figure 6), also depicted in a nomogram (Figure 7).

#### Predictive performance, accuracy, and clinical utility of the model

**Figure 8:**
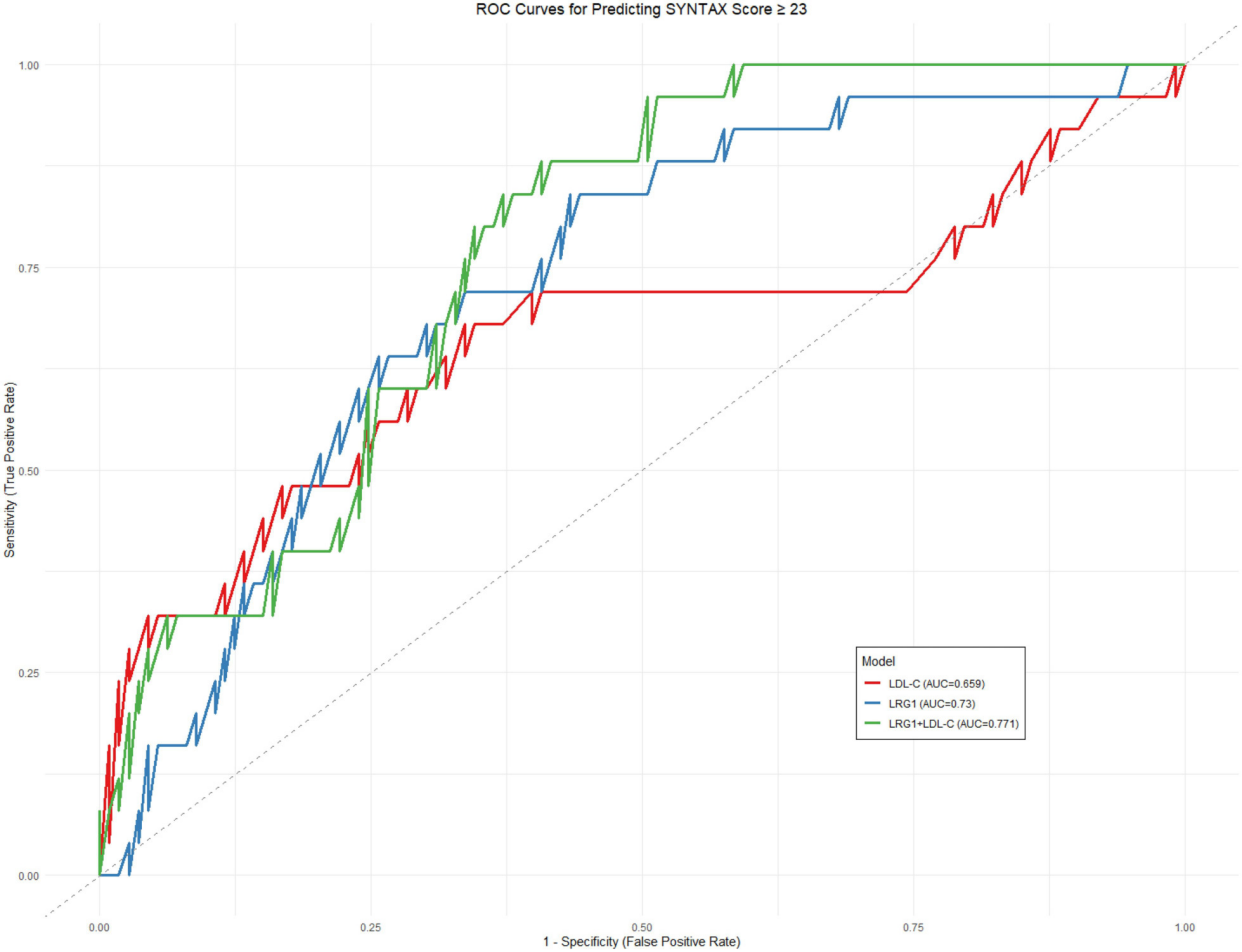
ROC curves for the predicting SYNTAX score ≥23 in CAD patients.

To assess the incremental predictive value of LRG1 beyond traditional lipid markers, the predictive performance of three nested models was compared. The AUC for LDL-C alone was 0.659 (95% CI: 0.518–0.801), the AUC for LRG1 alone was 0.73 (95% CI: 0.627–0.832), and the combined LRG1 + LDL-C model achieved an AUC of 0.771 (95% CI: 0.685–0.856). The combined model provided significantly better discrimination than LDL-C alone (DeLong test, *P* = 0.037). The addition of LRG1 to LDL-C resulted in a significant net reclassification improvement (categorical NRI = 0.723, *P* < 0.001) and an integrated discrimination improvement (IDI = 0.044, *P* = 0.058), confirming that LRG1 contributes predictive information that is complementary to LDL-C (Figure 8). The apparent C-statistic of the combined LRG1 + LDL-C model was 0.771 (95% CI: 0.685–0.856). After 1,000 bootstrap internal validation to correct for over-optimism, the bias-corrected C-statistic was 0.756, indicating minimal overfitting and confirming acceptable internal discriminatory performance.

**Figure 9:**
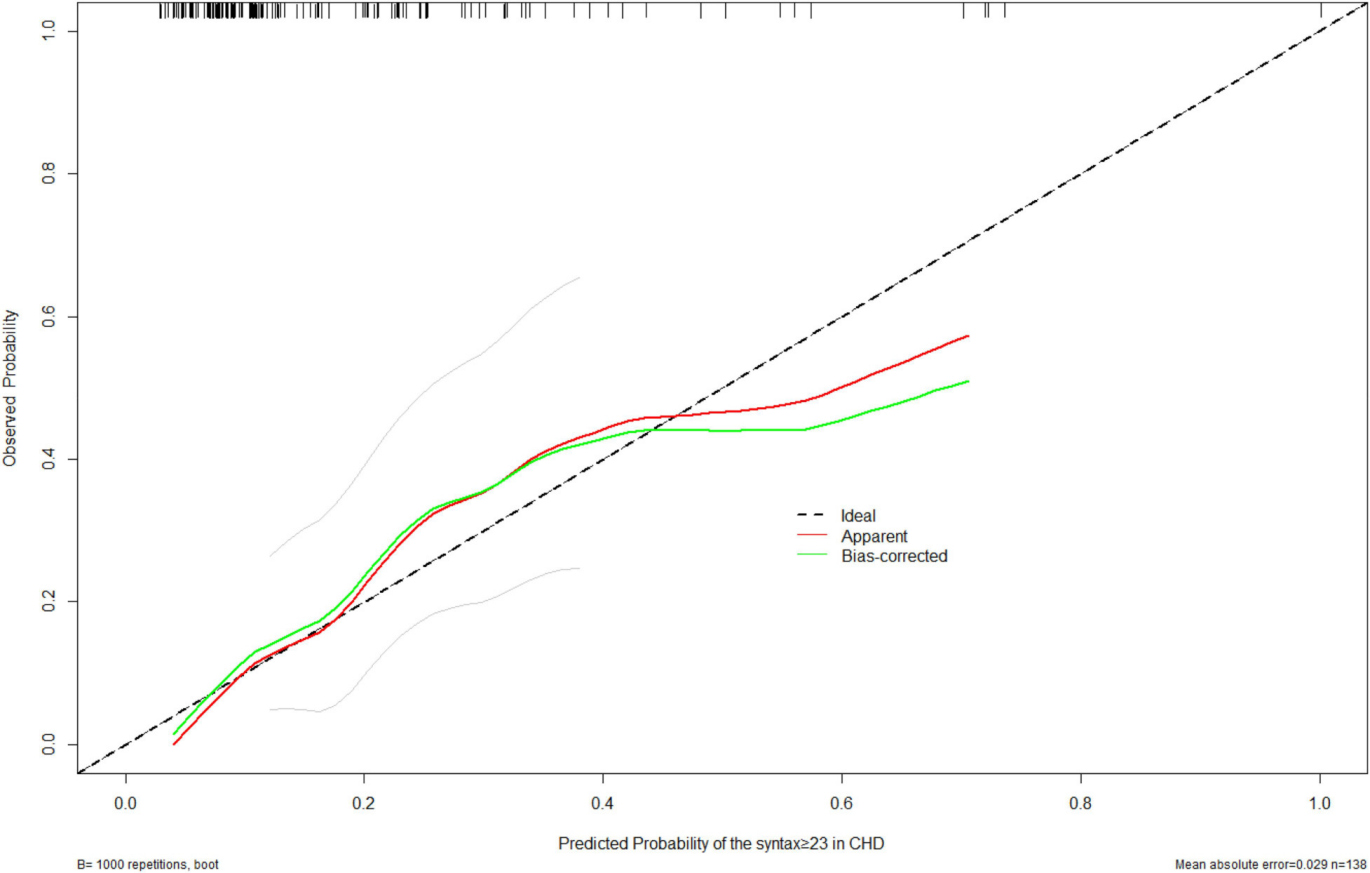
Calibration curves for predicting SYNTAX score ≥23 in CAD.

**Figure 10:**
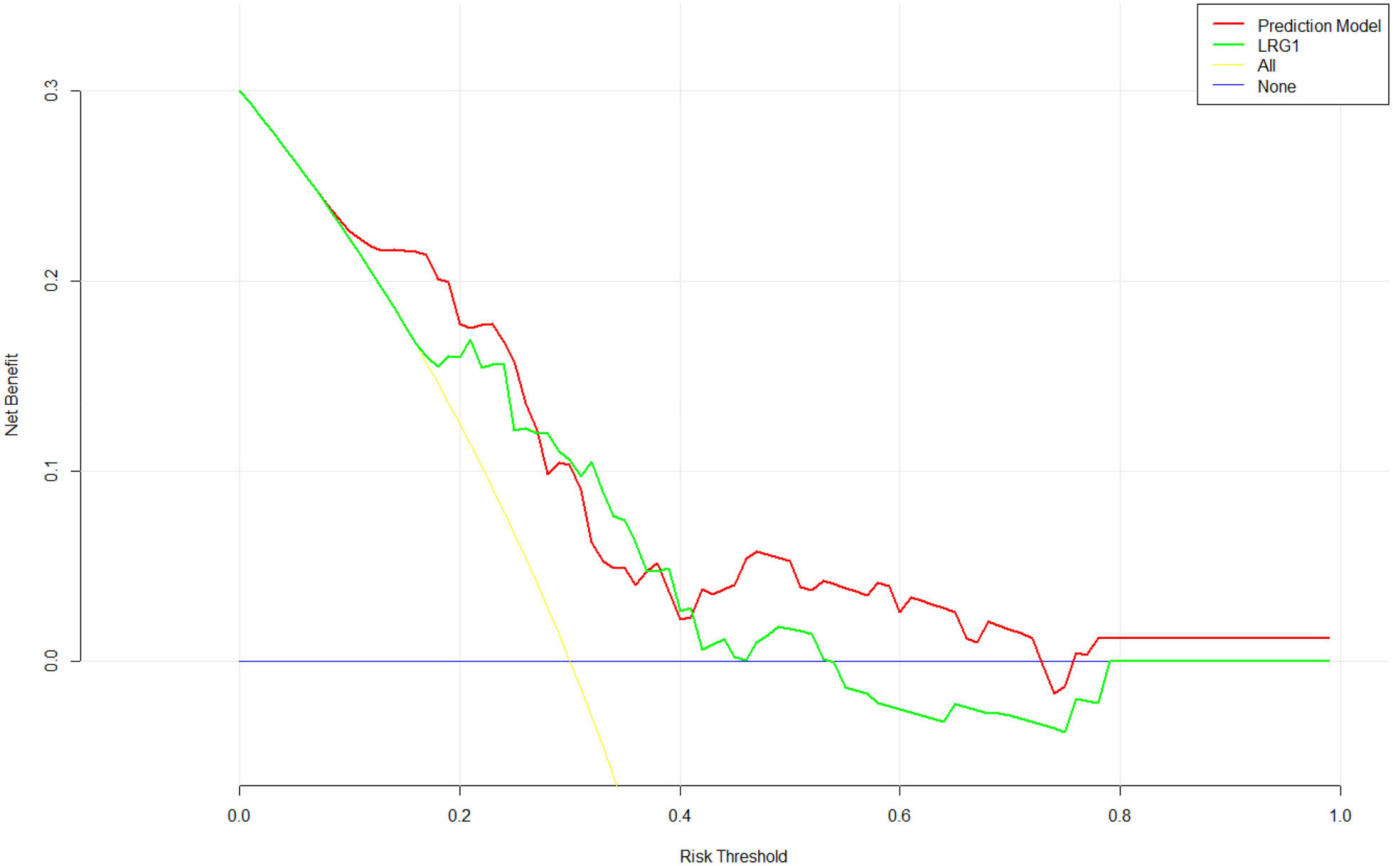
DCA of the prediction model, LRG1 for the SYNTAX score ≥23 prediction for CAD.

At the optimal cut-off value of 0.473 (based on the Youden index), the model achieved a sensitivity of 88.0% and a specificity of 59.3%. The Hosmer-Lemeshow test indicated excellent model fit (*χ*² = 8.18, degrees of freedom = 8, *p* = 0.416) (Figure 9). Decision curve analysis (DCA) demonstrated that the model offers greater net benefit, confirming its clinical utility (Figure 10).

## Discussion

Coronary artery disease (CAD) remains a predominant cause of mortality and morbidity globally, accounting for approximately seven million deaths annually (9). The pathological hallmark of CAD—coronary atherosclerotic lesions—critically influences patient prognosis and therapeutic decision-making. The SYNTAX Score, which quantifies lesion complexity, serves as a pivotal tool in determining the optimal revascularization strategy, distinguishing between coronary artery bypass grafting (CABG) and percutaneous coronary intervention (PCI) (10). Patients exhibiting a SYNTAX Score of 23 or higher are classified as having moderate to high CAD risk and demonstrate significantly elevated long-term incidences of major adverse cardiovascular events (MACE) and mortality relative to those with less complex lesions (11–14). Nevertheless, the reliance of the SYNTAX Score on invasive coronary angiography constrains its applicability for early, convenient, or dynamic risk stratification during the acute phase. Consequently, the identification of circulating biomarkers capable of non-invasive, pre-angiography screening of patients likely to harbor complex lesions is imperative. While coronary computed tomography angiography (CTA) has emerged as a valuable non-invasive imaging modality for assessing coronary stenosis and plaque burden, its widespread use as a first-line screening tool is constrained by cost, limited availability in resource-limited settings, radiation exposure, heart rate control requirements, and artifacts from severe coronary calcification. In contrast, serum biomarkers like LRG1 can be measured rapidly and inexpensively at the initial point of clinical contact—such as in the emergency department or outpatient clinic—without specialized equipment or radiation. The intended role of LRG1 is therefore not to replace CTA or invasive angiography, but to serve as a complementary pre-imaging triage tool that identifies patients with a high pre-test probability of complex coronary anatomy, thereby enabling more targeted and cost-effective use of subsequent imaging. Our study employs a two-step approach. First, publicly available transcriptomic data (GEO) were leveraged to demonstrate LRG1 upregulation in the acute phase of myocardial infarction. While these datasets used diagnostic classifications (AMI, SCAD), they provided a crucial molecular rationale. Subsequently, our clinical study was designed to address a more refined clinical question: whether circulating LRG1 levels are associated with the anatomical complexity of coronary lesions, regardless of the acute/chronic presentation, using the SYNTAX score as the gold standard. Given that coronary lesion complexity substantially dictates CAD prognosis (15), we assessed the predictive capacity of LRG1 for complex coronary anatomy. Muendlein et al. demonstrated that serum LRG1 is a predictor of all-cause mortality, vascular mortality, and MACE in patients undergoing coronary angiography (16). Our analysis revealed significantly increased LRG1 levels among patients with SYNTAX Scores ≥23. This indicates that LRG1 levels increase with the complexity of coronary artery lesions.

Through the application of Boruta feature selection, LASSO regression, and logistic regression analyses, LDL-C and LRG1 were identified as independent predictors of a SYNTAX Score equal to or exceeding 23. Consistent with its well-established role in atherogenesis (17–19), LDL-C reaffirmed its significance, while LRG1 provided additional prognostic information beyond traditional lipid parameters, highlighting the contribution of inflammatory mechanisms to lesion complexity. This incremental predictive value was formally demonstrated using the Net Reclassification Improvement (NRI) and Integrated Discrimination Improvement (IDI) indices. The addition of LRG1 to LDL-C resulted in a significant improvement in risk classification（NRI = 0.723, 95% CI: 0.319–1.127, *P* < 0.001), driven primarily by the correct downward reclassification of patients without complex lesions (NRI for non-events = 0.363, *P* < 0.001). The combined LRG1 + LDL-C model also achieved a statistically significantly higher AUC compared to LDL-C alone (DeLong test, *P* = 0.037). The IDI was 0.044 (95% CI: −0.001–0.090, *P* = 0.058), suggesting a trend toward improved overall discrimination that fell just short of statistical significance, possibly reflecting the limited number of outcome events. Collectively, these metrics confirm that LRG1 contributes meaningful and complementary predictive information beyond LDL-C alone, particularly in refining risk categorization. The association between LRG1 and coronary anatomical complexity is biologically plausible through several interrelated mechanisms. Beyond its role as a systemic inflammatory marker, LRG1 directly contributes to pathological angiogenesis within atherosclerotic plaques by modulating TGF-*β* signaling, thereby promoting intraplaque neovascularization, a hallmark of plaque instability and progression to complex, multi-vessel lesions. Recently, Wang et al. demonstrated that LRG1 drives pro-inflammatory macrophage M1 polarization, accelerating atherogenesis (20). This dual role in vascular inflammation and pathological vessel formation distinguishes LRG1 from generic acute-phase reactants and provides a mechanistic basis for its correlation with the anatomical complexity quantified by the SYNTAX Score, rather than solely reflecting myocardial necrosis or systemic inflammatory burden. Notably, LRG1 demonstrates a strong association with inflammatory mediators; its localized overexpression promotes neutrophil chemotaxis (21), which indirectly exacerbates vascular endothelial dysfunction. Furthermore, under inflammatory conditions, LRG1 directly impairs endothelial function by stimulating endothelial cell proliferation and capillary formation via activation of the TGF-*β* signaling pathway (3). The developed predictive model demonstrated robust statistical and clinical performance. This model surpassed the predictive capacity of individual risk factors, exhibited excellent calibration, and conferred substantial clinical net benefit. These findings suggest that the combined LRG1 and LDL-C panel, if externally validated, could serve as an adjunctive tool to support risk stratification and inform decisions regarding the urgency of invasive angiography.

In summary, addressing the clinical challenge of early, non-invasive identification of moderate to high-risk coronary lesions in CAD patients, this study builds upon prior evidence of elevated LRG1 expression in AMI. Our findings establish LRG1 and LDL-C as independent risk factors for complex coronary anatomy associated with adverse prognosis. The resultant predictive model demonstrated strong discriminative ability, reliable calibration, and tangible clinical utility. Nonetheless, several limitations of this study should be acknowledged. First, the sample size was limited, with 138 patients and only 25 outcome events (SYNTAX Score ≥23). Although the events-per-variable ratio (12.5) for the final two-predictor model met the minimum recommended threshold, the initial feature selection from 28 candidate variables using LASSO and Boruta may be unstable with this event rate, and the regression coefficients of LRG1 and LDL-C are likely inflated; consistent with this, the bootstrap-corrected C-statistic (0.756) was slightly lower than the apparent AUC (0.771), suggesting only minimal optimism. The borderline significance of the IDI (*P* = 0.058) may also reflect insufficient statistical power due to the modest event count. Second, the single-center retrospective design inherently limits generalizability; complete external validation in a prospective, multi-center setting is essential before any clinical application. Third, the study population included both AMI and non-AMI patients. As LRG1 is known to be elevated in acute myocardial infarction, heart failure, and renal dysfunction—conditions more prevalent in the high SYNTAX group—residual confounding by disease acuity cannot be excluded. Fourth, the model incorporated only two predictors, and other potentially relevant biomarkers or imaging variables were not considered. Fifth, the dichotomization of the SYNTAX Score at 23, while clinically meaningful, may lose prognostic granularity compared with the continuous score. Finally, LRG1 was measured using a research-grade ELISA; standardization across clinical laboratories and establishment of reference ranges are required before routine clinical use.

## Conclusions

In conclusion, this study provides preliminary evidence that serum LRG1, in conjunction with LDL-C, may serve as a non-invasive adjunct for identifying CAD patients with complex coronary anatomy (SYNTAX Score ≥23). If validated in larger prospective cohorts, this biomarker panel could facilitate earlier, non-invasive risk stratification and help identify patients who may benefit most from further anatomical evaluation.

## Data Availability

The original contributions presented in the study are included in the article/Supplementary Material, further inquiries can be directed to the corresponding author.
